# Rethinking Local Cancer Overscreening in Japan: A Path toward Organized Screening

**DOI:** 10.31662/jmaj.2025-0392

**Published:** 2026-02-20

**Authors:** Masanari Minamitani

**Affiliations:** 1 Department of Radiology, The University of Tokyo Hospital, Tokyo, Japan

**Keywords:** overscreening, organized screening, cancer screening, evidence-based policy, Japan

## Abstract

Japan’s cancer screening programs have expanded widely in recent decades, with local governments and workplaces offering screenings beyond national recommendations. Although this flexibility has improved access to screening, it has also contributed to overscreening and the practice of conducting tests beyond evidence-based age ranges, intervals, and methods. The underlying principles of organized screening, which emphasize evidence-based methods and quality assurance, have often been overshadowed by administrative and institutional momentum rather than by deliberate, evidence-based planning. In 2024, Miyazaki City initiated a comprehensive review of its cancer screening system under the new medical leadership. The process identified several deviations from national guidelines in target populations and screening methods, leading to a reform policy that discontinued non-evidence-based tests, such as the ABC method (a combination of serum pepsinogen and Helicobacter pylori antibody testing) for gastric cancer, breast ultrasound, and prostate-specific antigen testing. Although implementation is ongoing, this initiative demonstrates how municipalities can begin realigning screening practices with scientific standards. The Japanese case, exemplified by Miyazaki City’s initiative, highlights the importance of local leadership and an organizational understanding of the principles of organized screening. Reforming overscreening requires sustained collaboration between policymakers, healthcare providers, and citizens to balance accessibility with evidence-based practices.

Over the past few decades, health checkups and routine screenings have been accessible to almost all individuals throughout their lives in Japan ^(1)^. Local governments began cancer screening programs in the 1980s. Initially, local authorities systematically offered gastric and cervical cancer screening; over time, screening expanded to include lung, breast, and colorectal cancers. Although national guidelines specify the recommended methods, target ages, and intervals for these five types of cancer screening, local governments have maintained the authority to adjust programs according to regional circumstances ^(1)^. While this flexibility allows program tailoring to local needs and healthcare infrastructure, it also carries significant risks, including unnecessary procedures, psychological burden, and overdiagnosis. Benefits of screening are often overestimated, whereas the risks are underestimated ^(2)^. Consequently, many municipalities have implemented practices that constitute overscreening, defined as the use of screening tests outside the recommended age range or at a greater frequency than evidence-based guidelines suggest. More than 80% of local governments provided screening programs beyond the national recommendations ^(3)^.

Notably, this phenomenon was not confined to local communities. Workplace screening, which accounts for nearly half of all cancer screenings in Japan, involves a multi-stakeholder decision-making process involving personnel, labour affairs staff, occupational health professionals, and corporate executives ^(4)^. Even in these settings, screening is excessive, driven by the desire to offer employee welfare benefits rather than scientific evidence. Historically, Japanese companies have approached health provision with a paternalistic or familial mindset ^(5)^. Company-based cancer screening frequently exceeds national guidelines and varies according to company size, industry, and health policy ^(6)^.

The fundamental purpose of cancer screening is to reduce mortality from target cancers. Organized screening is the only established means of achieving this goal. The essence of organized screening lies in its implementation based on scientific evidence and in a robust quality assurance system. The tendency toward overscreening, which is common to both local governments and workplaces, is underpinned by the assumption that “routine cancer screening tests for healthy persons are almost always a good idea,” while the risks and limitations are overlooked ^(7)^. In such circumstances, from a utilitarian standpoint, over-servicing can occur easily when the perceived cost is reasonable ^(8)^. A lack of knowledge among decision-makers and practitioners regarding appropriate screening methods may create political, economic, and social incentives to override the scientific rationale. When deciding whether to initiate or discontinue a screening program, it is essential to consider local circumstances carefully, evidence supporting the effectiveness of screening, feasibility of changes, stakeholder support, political considerations, and values placed on prioritizing public health ^(9)^. In cases of non-recommended overscreening, strong resistance from stakeholders is likely, making it even more challenging to scale back or restore programs to appropriately organized screening ^(9)^.

In this context, Miyazaki City’s recent cancer screening reforms are noteworthy. In 2024, under the leadership of a newly elected mayor with a medical background, the city undertook a systematic review of its screening program. The city compared its existing cancer screening practices with national guidelines to assess their consistency with evidence-based recommendations. While follow-up pathways and quality assurance mechanisms were in place, several elements, such as target age ranges and test modalities, diverged from national standards. These findings, which were further compared with the WHO Screening Programmes: A Short Guide (summarized in Table 1), illustrate the challenges commonly observed in many Japanese municipalities, where screening programs have evolved incrementally without systematic evaluation against current evidence ^(9)^. Among these, deviations from national recommendations, particularly regarding target populations and overall processes, have become key concerns. Miyazaki City, therefore, established the Study Group for Optimizing Cancer Screening in Miyazaki City, comprising local healthcare providers, a public health official, and external academic experts, with municipal staff serving as secretariats. The group held four meetings between July 2024 and March 2025, and the final report was published on the municipality’s website on March 26, 2025.

**Table 1.** Comparison of Miyazaki City’s Cancer Screening Practices (as of 2024) with the WHO Screening Programmes: A Short Guide.

	Dimension	Status in Miyazaki City (as of 2024)	WHO requirement
1	Is the test carried out in isolation or is the test part of a pathway of care?	Positive cases can undergo further diagnostic procedures through Japan’s universal health insurance system.	The screening test is part of a pathway of care.
2	Who is eligible for screening?	Breast and gastric cancer screening provided to individuals outside national guidelines.	The eligible population is defined according to evidence, based on the balance of benefits versus harm.
3	How is the test offered?	Individual vouchers and reminders sent to non-attendees.	Systematically, based on a register of the eligible population using a call and recall system.
4	Is the pathway governed by protocols and guidelines?	Some examinations (gastric, breast, cervical, prostate) outside national recommendations.	Yes, decisions about an individual’s care is based on evidenced protocols and guidelines.
5	Are there quality standards based on evidence that are followed by screening providers?	General standards for organized screening in place.	All screening services within a screening programme agree on and use the standards.
6	Is the screening supported by an information system?	General information system established for organized screening.	Yes, an information system is in place linked to population registries.

Abbreviation: WHO, World Health Organization

The discussions addressed both general principles and site-specific issues across gastric, lung, colorectal, breast, cervical, and prostate cancer screening. The key themes included the role of organized screening as an evidence-based and quality-assuring system, the balance between benefits and harms, the feasibility of aligning local programs with national recommendations, and the need for effective communication with both citizens and screening providers. The group also emphasized strengthening collaboration with medical institutions to improve participation rates and maintain flexibility to adapt to revised national guidelines. As a result of these deliberations, the working group recommended several reforms, which included eliminating the ABC method (a combination of serum pepsinogen and Helicobacter pylori antibody testing) for gastric cancer, discontinuing ultrasonography for breast cancer, and removing prostate-specific antigen (PSA) testing for prostate cancer. Miyazaki City introduced breast ultrasound screening in 2005 due to limited mammography capacity. However, based on the current infrastructure and projected participation rates, the city concluded that mammography-based screening can now be adequately provided.

In Miyazaki City, with a population of approximately 400,000, the potential impact of discontinuing non-recommended cancer screening tests warrants careful consideration. For gastric cancer, the ABC method has no evidence of reducing mortality ^(10)^. Although its harms have not been conclusively quantified, concerns have been raised regarding high false-positive rates ^(10), (11)^. Similarly, ultrasound-only breast cancer screening is used in some low- and middle-income countries where mammography is difficult to provide ^(12)^. However, it has not yet been evaluated for mortality reduction or harm in high-quality comparative trials ^(12)^. For PSA-based prostate cancer screening, evidence is mixed. PSA-based prostate cancer screening can reduce mortality by approximately 1.3 per 1,000 men over 13 years, but it is also estimated that 20-50% of cancers detected through screening represent overdiagnosis ^(13)^. Screening recommendation levels vary across high-income countries, and men considering PSA-based prostate cancer screening should engage in individual decision-making discussions with their physician, weighing the benefits and harms of screening ^(13), (14)^. However, such an approach is difficult to implement within Japan’s current population-based screening framework, where routine invitation and limited individual consultation are standard.

Cost implications of discontinuing non-recommended screening tests can be estimated even if harms such as false positives or overdiagnosis cannot be quantified precisely. In Miyazaki City, the average annual participation over the past five years was approximately 5,500 for ABC screening, 15,500 for breast ultrasound cancer screening (with or without mammography), and 11,500 for PSA testing. The municipal subsidy per participant varied across screening pathways (group or individual), as well as by facilities and screening modalities, but was around 3,000 yen for ABC testing, 4,000 yen for breast ultrasound, and 1,000 yen for PSA screening. While a precise calculation is not possible, several tens of millions of yen in public funds were allocated annually to screening modalities with limited or uncertain evidence of effectiveness. Nationally, PSA testing was offered by approximately 80% of municipalities, and uterine cancer screening was provided by about 30% ^(3)^. Previous analyses estimated that overscreening occurs in 17% of municipalities for colorectal cancer, 50% for breast cancer, and 34% for cervical cancer ^(6)^. Given the widespread provision of non-recommended screening tests, these expenditures represent a substantial fiscal burden, particularly for local governments operating under constrained budgets. Although it was challenging to quantify individual-level harms such as false positives, overdiagnosis, or psychological distress from these non-recommended screening modalities, the combination of their large scale, uncertain benefit, and substantial fiscal burden highlights the need for policy attention.

The greatest challenge lies not in making policy decisions, but in their implementation. While policy decisions have been made within the city administration, their implementation is only beginning. Scaling back services long perceived as residents’ rights requires confronting the deeply rooted expectations of both residents and healthcare providers. Time is needed to assess how effectively these reforms can be realized, how stakeholders respond, and whether quality assurance is maintained after changes in service delivery.

As the Organization for Economic Co-operation and Development (OECD) has noted, Japan’s screening system faces structural challenges in balancing national standardization with local government autonomy ^(1)^. While some low-income countries report that expanding local decision-making authority can empower political actors and increase the likelihood of low-value interventions ^(15), (16)^, Japan’s situation, in which most municipalities provide screening programs that diverge from national recommendations in target groups, intervals, and methods, is unique among high-income nations ^(1)^. Thus, the Miyazaki City reform represents a preliminary example of whether excessive screening systems in Japan can be restructured into appropriate, organized screening at the municipal level. Although the outcomes are yet to be observed, the Miyazaki case illustrates that evidence-informed reform processes can emerge even within deeply institutionalized local systems.

## Article Information

### Acknowledgments

The author is grateful to the members of the Study Group for Optimizing Cancer Screening in Miyazaki City and the Miyazaki City Office, and the public health center staff. The author also wishes to thank Dr. Hiroshi Saito for his constructive feedback and insightful suggestions during the manuscript revision process.

### Author Contributions

The author solely conceived and drafted the manuscript.

### Conflicts of Interest

The author was a member of the Study Group for Optimizing Cancer Screening in Miyazaki City.
